# Violence Exposure and Cognitive Outcomes Among Children in Low- and Middle-Income Countries (LMICs): A Systematic Review

**DOI:** 10.1177/15248380251316232

**Published:** 2025-03-12

**Authors:** Lucinda P. Tsunga, Lucy V. Hiscox, Sarah L. Halligan, Kirsten A. Donald, Abigail Fraser

**Affiliations:** 1 University of Bristol, UK; 2 University of Cape Town, South Africa; 3 Cardiff University, UK; 4 University of Bath, UK; 5 Stellenbosch University, Cape Town, South Africa

**Keywords:** violence exposure, war, domestic violence, child abuse

## Abstract

This article examines how exposure to violence in childhood is linked to impaired cognitive functioning and academic performance. Children who reside in low- and middle-income countries (LMICs) are more likely to be exposed to violence yet their representation in published studies is often limited. Here, we conducted a systematic review to examine the evidence regarding the association between childhood violence exposure and cognitive outcomes assessed up to age 11 in children from LMICs. EMBASE, Medline, and PsychInfo were systematically searched to identify cross-sectional, case-control, or cohort studies published from inception to May 2023. All studies were assessed for risk of bias. We identified 17 studies that met our inclusion criteria, encompassing 27,643 children from 20 LMICs. Children were exposed to maltreatment, intimate partner violence, and war. Cognitive outcomes assessed included cognitive development (*n* = 9), executive functioning (*n* = 6), general intelligence (*n* = 2), language (*n* = 2), and memory (*n* = 1). A majority (71%) of the studies found a relationship between violence exposure and poor cognitive outcomes in childhood. Our findings suggest associations between different forms of violence and poor cognitive outcomes in children in LMICs. An increased investment in prevention is needed to tackle this human rights violation, and early interventions are important to ensure that LMIC children achieve their full potential. This is crucial in LMICs in which the burden of violence is high.

## Background

Exposure to violence during childhood is a significant human rights and public health concern that can result in enduring adverse health and social outcomes throughout an individual’s life. To date, the existing literature has primarily focused on investigating the effects of violence exposure on physical health (see reviews: Suglia et al., 2015; Wright et al., 2016) or mental health and psychosocial functioning (see reviews: Carr et al., 2020; Fong et al., 2019; Fowler et al., 2009). Cognitive outcomes have received comparatively less attention. Cognition plays a role in emotional processing, social functioning, educational achievement, and vocational outcomes (Perkins & Graham-Bermann, 2012). As such, cognitive impairments can have far-reaching consequences, significantly affecting a child’s day-to-day functioning and long-term prospects (Perkins & Graham-Bermann, 2012).

The available research investigating the potential impact of childhood exposure to violence on cognitive outcomes has predominantly been conducted in adolescents and adults living in high-income countries (HICs, see reviews: Matte-Landry et al., 2022; Savopoulos et al., 2023; Young-Southward et al., 2020). Overall, studies have described associations between maltreatment in childhood and poor cognitive outcomes, including lower general intelligence, poorer literacy and numeracy skills, and deficits in executive functions such as attention and working memory (see reviews: Su et al., 2019; Young-Southward et al., 2020). Associations have also been found between exposure to intimate partner violence (IPV) and poorer intelligence quotient (IQ) scores, poor verbal abilities, and academic skills (Savopoulos et al., 2023). While there is limited research focusing on community violence, one study conducted in the United States found that adolescents exposed to community violence were at risk for lower IQ scores (Butler et al., 2018). Other research found that U.S. pre-schoolers living in violent contexts exhibited attention problems, poor impulse control, and lower pre-academic skills (McCoy et al., 2015; Sharkey et al., 2012). There is also a link between impaired cognitive functions, such as attention and executive control, and mental health problems such as anxiety, depression, and PTSD, often creating a feedback loop that worsens emotional regulation and social functioning (McCrory et al., 2010). Chronic stress and adversity, such as violence, disrupt neurodevelopment, increasing vulnerability to lifelong mental and physical health issues (Shonkoff et al., 2012).

It is well-documented that children in LMICs have a greater exposure to violence than those in HICs (Butchart et al., 2015). The added burden of violence is taxing on LMICs, which have developing economies and under-resourced public health systems (Pereznieto et al., 2014). Furthermore, the impact of violence exposure on children’s cognitive abilities, and in turn educational outcomes, may have socioeconomic consequences at both the individual and societal levels (Perkins & Graham-Bermann, 2012). Understanding the potential consequences of violence exposure for cognitive outcomes in children living in LMICs, and the factors that could mitigate against those consequences, can inform policy and interventions to prevent persistent adverse consequences of violence exposure (Perkins & Graham-Bermann, 2012), and potentially contribute to breaking cycles of violence and poverty (Walker et al., 2011).

Violence in LMICs manifests in distinct patterns based on type and perpetrator. *IPV* is predominantly physical and sexual, with men as primary perpetrators against women (Devries et al., 2013). *Child abuse* includes physical abuse, maltreatment, and sexual abuse, mainly occurring at home or in schools, with parents or caregivers as key perpetrators (Mercy et al., 2017). *Community violence*—encompassing gang violence, street crime, and youth violence—is driven by poverty, unemployment, and inequality, often affecting children and youth in urban areas with weak infrastructure and law enforcement (Mercy et al., 2017). In 2011, the interpersonal violence death rate was 8.0 per 100,000 in LMICs, compared to 3.3 per 100,000 in HICs (Mercy et al., 2017). *Collective violence*, including war, political violence, and terrorism, is fueled by political instability, ethnic tensions, and resource competition, leading to displacement and regional destabilization (Elfversson, 2021; Le et al., 2022).

We conducted a systematic review and synthesized evidence that examined associations between exposure to various forms of violence in childhood and cognitive outcomes in children residing in LMICs. Our specific research questions were: is children’s exposure to violence in LMICs associated with their cognitive performance and is there any evidence of potential moderators of violence-cognition associations?

## Methods

### Protocol

The process and reporting of results in this systematic review were guided by the 2020 PRISMA statement for reporting systematic reviews (Page et al., 2021). The protocol was registered on PROSPERO (CRD42021268450, 25 August 2021).

### Eligibility Criteria

We included observational studies that investigated linkages between childhood violence exposure and cognitive outcomes assessed in children aged 11 or younger. The studies included comparisons between violence-exposed and non-exposed groups, as well as investigations into the associations between the extent of violence exposure and cognitive functioning. Study designs included cross-sectional, case-control, and cohort studies. There were no restrictions on publication dates. All studies had to be written in English due to the authors’ language constraints. Reviewed studies were those published in peer-reviewed journals as well as the grey literature.

The target population comprised children living in LMICs. The Organization for Economic Co-operation and Development (OECD) classification system was used to classify countries as LMICs based on income levels and economic development (see Supplemental Table 1). LMIC status of countries was based on their classification at the time of the search rather than the time of data collection. This maintained consistency across the studies and aligned with current socioeconomic classifications. Studies were included if the mean age of participants at the outcome assessment was 11 years or under or if at least 80% of the sample fell within this age range. This age range captures key stages of cognitive development in childhood. By including children up to 11 years, we encompassed both early and middle childhood, ensuring a comprehensive assessment of cognitive outcomes related to exposure to violence during these critical developmental stages.

Definitions of the types of violence considered are presented in Table 1. Based on existing characterizations (Edleson, 1999; Gredler 2003; Jouriles et al. 2001; Krug & World Health Organization, 2002; Wolak & Finkelhor 1998; World Health Organization 1999) acts of violence could include: (a) violence of a sexual nature, such as unwanted touching, forced sex, attempted unwanted sex, sexual harassment, or pressurized/coerced sex), (b) emotional acts of violence, such as verbal and psychological abuse, (c) physical acts of violence, such as corporal punishment, violent discipline, and physically abusive behaviours, (d) neglect, (e) bullying, such as cyber, physical, or verbal bullying, (f) witnessing domestic violence or parental IPV, (g) witnessing community violence, including sexual assault, burglary, mugging, the sound of gunshots, and gang violence, (h) collective violence committed by larger groups of individuals or by states, including social, political, war, and economic violence.

**Table 1.** Childhood Violence Exposure Definitions.

Physical violence/abuse	Acts that involve inflicting physical harm or having the potential to cause harm. These acts are typically under the control of a parent or an individual in a position of responsibility, power, or trust. They may occur as isolated incidents or be repeated over time.
Sexual violence/abuse	Instances where a child is engaged in sexual activity without full comprehension, the ability to give informed consent, or the necessary developmental readiness. It encompasses activities that violate both legal and social norms within society. Child sexual violence can involve an adult or another child who, due to age or developmental differences, holds a position of responsibility, trust, or power. The purpose of such activity is to fulfill the needs or gratify the other individual involved.
Emotional violence/abuse	The failure to provide a nurturing and appropriate environment including the availability of a primary attachment figure supports a child’s development of emotional and social competencies in line with their potential and societal context. It involves acts that have the potential to cause harm to the child’s health, physical, mental, spiritual, moral, or social development. These acts are typically within the control of a parent or an individual in a position of responsibility, trust, or power. Examples of such acts include restricting the child’s movement, engaging in patterns of belittling, denigrating, scapegoating, threatening, scaring, discriminating, ridiculing, or employing other non-physical forms of hostile or rejecting treatment. The ultimate effect is hindering the child’s ability to develop a stable and comprehensive range of emotional and social skills.
Neglect	The failure to adequately meet the child’s needs across various domains, including health, education, emotional development, nutrition, shelter, and safety, taking into account the available resources accessible to the family or caretakers. It involves acts or omissions that have the potential to cause harm to the child’s health, physical, mental, spiritual, moral, or social development. This includes the failure to provide appropriate supervision and protection to children to the extent that is reasonably feasible, ensuring their well-being and safety.
Witnessing domestic violence	When children see, hear, actively intervene in, or personally experience the consequences of physical or sexual assaults involving their caregivers.
Bullying	Bullying involves enduring repeated negative actions from one or more individuals over an extended period. The victim often faces challenges in defending themselves against such behavior. The review will include studies on both the perpetration and victimization of bullying, including cyberbullying and peer-to-peer victimization.
Community violence	Children’s exposure to interpersonal violence outside of their homes, schools, institutions, or organized workplaces can occur through witnessing, perpetrating, or being directly victimized. This form of violence, known as community violence, encompasses various types such as physical violence, sexual violence, assaults by authority figures (e.g., police), and violence linked to gangs and traffickers.
Collective violence	Violence perpetrated by larger groups or states can be classified into different categories. Social violence refers to violence carried out to promote a specific social agenda. Political violence encompasses acts associated with war, violent conflicts, state violence, and similar actions conducted by larger groups. Economic violence involves attacks motivated by economic gain, orchestrated by larger groups.

*Note*. The definition of physical, sexual, and emotional violence and neglect is from the World Health Organization (1999). The definition of domestic violence is from (Edleson, 1999; Jouriles et al., 2001; Wolak & Finkelhor, 1998). The bullying definition is from Gredler (2003). Community and collective violence as defined by the World Report on Violence and Health, World Health Organization (Krug et al., 2002).

Cognitive function encompasses a wide range of mental processes and abilities that enable individuals to acquire knowledge, process information, and engage in reasoning. Domains of cognitive functions include executive functioning (attention, working memory, inhibitory control, problem-solving, abstraction, planning and organization, cognitive flexibility); learning and memory (verbal, visual, tactile, prospective, remote memory), language ability (expressive, receptive language), intelligence (IQ, reasoning), processing speed, perception and motor functions, social cognition and academic performance (Kiely, 2014; Palmese, 2017). We also included interrelated developmental aspects such as motor function and socioemotional development where they were investigated within a cognitive development framework (Gandotra et al., 2023). This was based on an understanding of the interdependence of these groups of functions. For example, motor skills enhance cognitive growth through exploration and spatial learning, while socio-emotional development supports executive functions such as self-regulation and attention (Gandotra et al., 2023).

### Exclusion Criteria

Exclusion criteria were: (a) Studies that examined children with special conditions, including disability or serious mental illness; (b) Studies that measured violence exposure indirectly such as classifying communities as violent without directly reporting individual exposure. However, studies on IPV were included even when there were no indices capturing whether children witnessed said violence, given that young children are often in their homes/the presence of their caregivers and are therefore particularly likely to be exposed to IPV (Fantuzzo et al., 1997; Kitzmann et al., 2003).

### Information Sources and Search Strategy

A comprehensive search was conducted in EMBASE, Medline, and PsycINFO using the search terms listed in Table 2. We identified key search terms related to violence exposure, cognitive outcomes, and childhood as used in previous reviews (Su et al., 2019; Young-Southward et al., 2020). We combined these terms using Boolean operators to create a comprehensive search string (see Supplementary Table 2 for the full search strategy and Supplementary Table 2). The reference lists of the included studies were also examined to identify any additional relevant articles. Grey literature in the form of dissertations and theses uncovered through database searches were also included to reduce publication bias. Searches were initially conducted in 2021 and then updated in May 2023.

**Table 2.** Description of Selected Studies, Including Participant Characteristics, Violence Exposure Types, Cognitive Outcomes Measures, and Main Study Findings.

Study Characteristics		Participant Characteristics			Violence Exposure			Cognitive Outcome(s)	
Study (Year), Country	Study Design	Sample Size	Age(s)	Sex	Violence Exposure Type(s); Reporter	Age at Exposure	Tool	Type; Reporter	Measure
**Barnett et al. (2021)**, South Africa	CS	626	2 years	Males and Females	Domestic Violence (Maternal emotional, physical and sexual IPV); Mothers	Within the last 12 months prior to the study	IPVQ	Neurodevelopment; Children	BSID-III
Barrera et al. (2013), Colombia	CS	76	Means Controls: 10.11, Cases: 10.23, PTSD+: 10.92, PTSD–: 9.88 years	Males and Females	Maltreatment (sexual abuse), N/A	Not reported	None	Executive functioning Attention Memory Visual Perceptual and Constructional abilities Motor ability; Children	TMT, CVLT, ROCFT, Stroop Color-Word Interference Test, WCST
Bengwasan and Bancual (2020), Philippines	CS	206	3–12 years	Males and Females	Maltreatment: physical abuse, sexual abuse, neglect; Social Workers	At least 6 months prior to the study	None	Cognitive Development (Intellectual abilities) Communication (expressive and receptive communication skills); Parents, Caregivers, or Guardians	DP-3
Bernardes et al. (2020), Brazil	CS	2016	Mean 9.72 (6–12) years	Males and Females	Maltreatment (emotional and physical neglect, and emotional, physical, and sexual abuse); Children and Caregivers	Lifetime exposure	CTQ	Executive functioning; Children	WISC III, Corsi blocks, M-Stroop, Go/NoGo, ROCF, BQSS, Savage system
Carvalho et al. (2017), Brazil	CS	85	Control Group Mean 9.53 years, Maltreatment Group Mean 9.64 (6–12) years	Males and Females	Maltreatment; Children and Caregivers	Exposure to maltreatment prior to institutionalization, specific age not reported	JVQ	General intellectual Functioning abilities, Attention and Working Memory; Children	WASI, WISC III, WISC-IV subtests
**Diab et al. (2018)**, West Bank and Gaza Strip	CS	303	Mean 10.94 (10.3–13.5) years	Males and Females	War Violence; Children	Not reported	Gaza Traumatic Event Checklist	Academic achievement (language and math scores); Children	Two examinations
Hecker et al. (2016), Tanzania	CS	409	Mean age 10.50 (6–15) years	Males and Females	Maltreatment (Corporal punishment); Children	Lifetime exposure	pediMACE	School performance and Working Memory capacity; Children	Mathematics, English, Swahili, Science, Corsi blocks
Jeong et al. (2020), Benin, Cambodia, Cameroon, Democratic Republic of the Congo, Honduras, Jordan, Rwanda, Senegal, Timor-Leste, Togo	CS	15202	Mean age 47.22 (36–59) months	Males and Females	IPV; Mothers	Within the last 12 months prior to the study	CTSPC	ECD; Mothers	ECDI
Julio et al. (2023), Philippines	CS	1506	10–12 years	Males and Females	Domestic Violence: physical IPV, emotional or psychological IPV, and controlling behavior; Mothers	Lifetime exposure and exposure in the last 12 months prior to the study	CLHNS	Mathematical Ability, English Reading Skills, Native Language Reading Ability, and Nonverbal Intelligence; Children	CLHNS, Philippine nonverbal intelligence local test
Kohrt et al. (2015), Peru	CS	97	Mean 8.24 (5–11) years	Males and Females	Domestic Violence; Mothers	Lifetime exposure	WAST	Cognitive development; Children	Batería III
Leyton (2020), Honduras	CS	2256	3–4 years	Males and Females	Domestic Violence (IPV- controlling, emotional, physical and sexual violent behaviors); Mothers	Lifetime exposure	CTS	ECD; Mothers	ECDI
Malik et al. (2010), Pakistan	CS	100	Mean 10.38 (8–12) years	Males and Females	Maltreatment; Children	Lifetime exposure	CAS	Cognitive deficits and Reading problems; Teachers	CBRSC
Rocha et al. (2021), Brazil	CS	3566	31.8 months	Males and Females	Domestic Violence (IPV); Mothers or head of the household	Within the last 12 months prior to the study	HITS	Child development; Mothers	ASQ-BR
Sartori et al. (2017), Brazil	CS	82	8–9 years	Males and Females	Maltreatment (parental neglect) and Domestic violence (physical or sexual); Social Workers	Exposure to maltreatment before institutionalization, specific age not reported	None	Motor ability; Children	MABC-2
Vameghi et al. (2016), Iran	CS	750	10.53 (6–18) months	Males and Females	Domestic Violence (physical, sexual, or emotional violence); Mothers	Lifetime exposure	Domestic Violence Questionnaire	Cognitive Development; Mothers	ASQ
Xing & Wang (2018), People’s Republic of **China**	CS	150	Mean 10.83 (9–11) years	Males and Females	Maltreatment (Corporal punishment); Children	Within the last 12 months prior to the study	CTSPC	Executive Function; Mothers and Fathers	BRIEF-Parent Form
Xing et al. (2019), People’s Republic of China	L	213	Mean 4.80 years	Males and Females	Maltreatment (Corporal punishment); Mothers and Fathers	Corporal punishment within the past 12 months, measured a year prior cognitive outcomes	CTSPC	Executive Function; Children	House and Pick the Picture, Something is the Same, Arrow, Pig, and Silly Sounds Game score

*Note*. ASQ = Ages and Stages Questionnaire; ASQ-BR = Ages and Stages Questionnaire–Brazil; ADHD = attention deficit hyperactivity disorder; Batería III = Woodcock-Johnson III—Spanish Version; BQSS = Boston Qualitative Scoring System; BRIEF = Behavior Rating Inventory of Executive Function, Second Edition; BSID-III = Bayley Scales of Infant and Toddler Development, third edition; CAS = Child Abuse Scale; CBRSC = Comprehensive Behavior Rating Scale for Children; CDC = Center for Disease Control; CLHNS = Cebu Longitudinal Health and Nutrition Survey; CTQ = Childhood Trauma Questionnaire; CTS = Conflict Tactics Scale; CTSPC = Parent-Child Conflict Tactics Scale; CS = cross-sectional; CVLT = California Verbal Learning Test; DP-3 = Developmental Profile III; ECD = early child development; ECDI = early child development index; HITS = Hurt, Insult, Threaten, Scream Questionnaire; IPV = intimate partner violence; IPVQ = Intimate Partner Violence Questionnaire; JVQ = Juvenile Victimization Questionnaire; L = longitudinal; M-Stroop = modified form of stroop; MACE = maltreatment and abuse chronology of exposure—Pediatric Version; MABC-2 = movement assessment battery for children, 2nd Edition; PTSD = post-traumatic stress disorder; ROCFT = Rey-Osterrieth Complex Figure Test; TMT = Trail Making Test; TOL = Tower of London Test; WASI = Wechsler Abbreviated Scale of Intelligence; WASI-II = Wechsler Abbreviated Scale of Intelligence, Second Edition; WCST = Wisconsin Card Sorting Test; WAST = Woman Abuse Screening Tool; WISC III = Wechsler Intelligence Scale for Children, Third Edition; WISC-IV = Wechsler Intelligence Scale for Children, Fourth Edition; WRAT = Wide Range Achievement Test.

### Data Management and Selection

Title and abstract screening were conducted by one reviewer (L.P.T.). Full-text screening of the articles deemed eligible for inclusion was conducted by two independent reviewers (L.P.T. and L.V.H.), with 90% interrater reliability. Reviewers initially differed regarding the inclusion/exclusion of four articles and disagreements were resolved through discussion.

### Data Extraction

Data on authors, publication year, study design, country of study, participant characteristics, (i.e., sample size, age, sex), violence exposure (type, measurement tool, respondents), cognitive outcomes (type, measure) and key results were extracted by two independent reviewers (L.P.T. and L.V.H.).

### Risk of Bias Assessment

Two independent reviewers (L.P.T. and L.V.H.) used the Risk of Bias Assessment tool for Non-randomized Studies (RoBANS; Park et al., 2011) to assess the risk of bias. Any conflicts were addressed and settled by consensus. Risk of bias was assessed according to the six domains of the RoBANS: (a) selection of participants; (b) confounding variables; (c) measurement of exposure; (d) blinding of outcome assessments (applied to case-control studies); (e) incomplete outcome data; and (f) selective outcome reporting. Risk of bias was rated as *low*, *high*, or *unclear* for each domain.

#### Confounding Control Assessment

Based on previous literature, we compiled a list of key confounding variables that may influence the relationship between violence exposure and cognitive outcomes in childhood. Eight confounding variables were identified and grouped into three domains: Sociodemographics (child age, child sex); Socioeconomics (household income, parental education); and Caregiver Characteristics (alcohol use, other substance use, and mental health status). Each of these factors can confound the relationship between violence exposure and cognitive outcomes in children, i.e. may be associated with both the exposure and the outcome (VanderWeele, 2019). Specifically, children’s exposure to violence and cognitive abilities may vary with age (Gilmore et al., 2018; Tideman & Gustafsson, 2004). Boys and girls may experience and respond to violence differently due to biological and social factors (Kheloui et al., 2023). Parental SES and education impact access to cognitive stimulation and educational resources, while also increasing the likelihood of violence exposure (Hertzman & Boyce, 2010; Osler et al., 2013). Caregiver factors such as alcohol and substance use impair caregiving capacity, heighten exposure to violence, and directly affect child cognitive development through neurotoxic effects (Hendricks et al., 2020; Morie et al., 2019; World Health Organization, 2020). Caregiver mental health, particularly depression, disrupts parenting quality and increases household violence, compounding its effects on child cognition (Conway et al., 2020).

We classified each confounder as being “adequately” versus “inadequately” controlled for based on whether or not it was adjusted for in the design or analysis. Adjustment for potential confounding was assessed as: “adequate control”—when at least one variable from each construct was adjusted for; “inadequate control”—a lack of adjustment for any variable in any construct; or “some concerns”—when adjustment for at least one variable was made for some, but not for all, constructs. We used the R package *metaconfoundr* to visualize the adequacy of adjustment for confounding variables by creating a confounding matrix (Figure 6; Petersen et al., 2022).

### Data Synthesis

The forms of violence and cognitive outcomes assessed in each of the reviewed studies varied. Variation in the types of violence captured resulted in fewer than five unique studies with exposure measurements that were deemed sufficiently similar to pool. We therefore did not conduct a meta-analysis and instead provided a descriptive synthesis.

## Results

**Figure 1.**
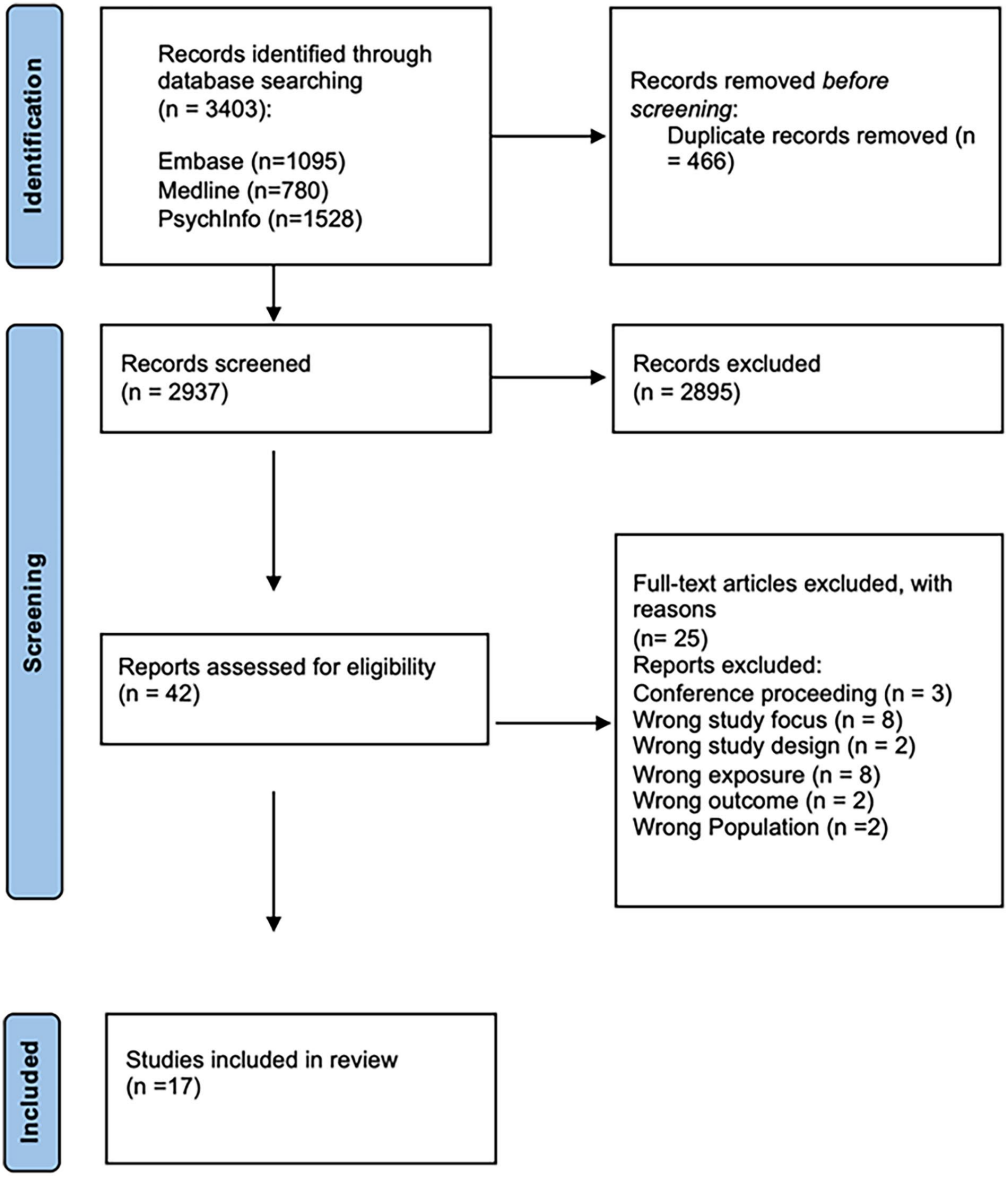
PRISMA flow diagram showing manuscript selection.

A total of 3,403 records were retrieved. Removal of duplicates resulted in 2,937 records for title and abstract screening. This screening excluded a further 2,895 records, leaving 42 articles for full-text screening. From these, a further 25 studies were excluded at this stage as they did not meet the study’s inclusion criteria (see Figure 1). The final number of studies qualitatively synthesized in this review was *n* = 17.

### Description of Included Studies

Table 2 summarizes the key characteristics of the 17 included studies. All studies were published in peer-reviewed journals between 2010 and 2023. Sixteen studies were of a cross-sectional design in which violence exposure was retrospectively reported. One study had a longitudinal design with violence exposure assessed at an average age of 4.8 years, 1 year prior to the assessment of cognitive outcomes.

The total number of participants (*N*) within these studies ranged from 76 to 15,202 with a median of 303. The total *N* for the review is 27,643. The age of the samples ranged from 2 to 15 years, with an overall mean age of 8 years. All 17 of the studies included both male and female participants. The reviewed sample largely consisted of community samples; only one study included institutionalized children.

Studies were from 20 LMICs in total, spanning four continents (Africa, Asia, North America, and South America): One study was a multi-country study conducted in Benin, Cambodia, Cameroon, the Democratic Republic of the Congo, Honduras, Jordan, Rwanda, Senegal, Timor-Leste and Togo. The remaining studies were single-country studies: four studies were based in Brazil, two studies each were from the People’s Republic of China and Philippines, and the remaining eight studies were conducted in Columbia, Honduras, Iran, Pakistan, Peru, South Africa, Tanzania, and West Bank and Gaza.

### Violence Exposure

The majority of studies (*n* = 9) investigated child maltreatment (Barrera et al., 2013; Bengwasan & Bancual, 2020; Bernardes et al., 2020; Carvalho et al., 2017; Hecker et al., 2016; Malik et al., 2010; Sartori et al., 2017; Xing & Wang, 2018; Xing et al., 2019). Seven studies investigated exposure to IPV (Barnett et al., 2021; Jeong et al., 2020; Julio et al., 2023; Kohrt et al., 2015; Leyton, 2020; Rocha et al., 2021; Vameghi et al., 2016), and one study investigated war violence (Diab et al., 2018).

### Outcomes

A range of cognitive outcomes were investigated (see Table 2). Domains covered were early childhood cognitive, socioemotional and motor development (nine studies: Barnett et al., 2021; Barrera et al., 2013; Bengwasan & Bancual, 2020; Jeong et al., 2020; Julio et al., 2023; Leyton, 2020; Rocha et al., 2021; Sartori et al., 2017; Vameghi et al., 2016); executive functioning, including attention and working memory (six studies: Barrera et al., 2013; Bernardes et al., 2020; Carvalho et al., 2017; Hecker et al., 2016; Xing & Wang, 2018; Xing et al., 2019); general intelligence (three studies: Carvalho et al., 2017; Julio et al., 2023; Malik et al., 2010); language ability (two studies: Malik et al., 2010; Rocha et al., 2021); academic achievement (two studies: Diab et al., 2018; Hecker et al., 2016); and memory (Barrera et al., 2013). Numerous cognitive instruments were used to assess cognition (see Table 3).

**Table 3.** Results of Studies Not Included in Forest Plots and Studies with Mediation and Moderation Analyses.

Study Characteristics		Participant Characteristics			Violence Exposure			Cognitive Outcome(s)		Results
Study (Year), Country	Study Design	Sample Size	Age(s)	Sex	Type(s); Reporter	Exposure Age	Tool	Type; Reporter	Measure	
Bengwasan and Bancual (2020), Philippines	CS	206	3–12 years	Males and Females	Maltreatment: physical abuse, sexual abuse, neglect; Social Workers	At least 6 months prior to the study	None	Cognitive Development (Intellectual abilities) Communication (expressive and receptive communication skills); Parents, Caregivers, or Guardians	DP-3	Cognitive scores (M = 80.971, SD = 16.931), and Communication scores (M = 76.660, SD = 18.703) were significantly lower than the minimum Average Standard Score, *p* < .001. There were significant differences between the three types of abuse in relation to the different DP-3 areas, Wilks’ Lambda = 0.868, *F*(10, 398) = 2.916, *p* < .01: Sexually abused (M = 81.356, SD = 18.020) and physically abused (M = 79.302, SD = 16.674) groups had a significantly higher mean score in Communication compared to neglected group (M = 69.386, SD = 16.766), *F*(2, 203) = 21.604, *p* < .001 with a moderate effect size, partial η^2^ = .080. For Cognitive Development, the sexually abused group (M = 85.699, SD = 16.415) had a significantly higher mean score than the neglected group (M = 75.671, SD = 17.213), *F*(2, 203) = 21.682, *p* < .001 with a moderate effect, partial η^2^ = .061
Diab et al. (2018), West Bank and Gaza Strip	CS	303	Mean 10.94 (10.3–13.5) years	Males and Females	War Violence; Children	Not reported	Gaza Traumatic Event Checklist	Academic achievement (language and math scores); Children	Examinations	The direct effects model showed that exposure to war violence was not significantly associated with children’s low academic achievement (β = –.05, *t* = –0.74). Parental scholastic involvement and children’s motivation and learning strategies mediated between war violence and academic achievement (β =* −*.21, *t* = –2.60, *p* < .009): High exposure to war violence was associated with lower levels of encouraging scholastic involvement from parents, which in turn was significantly associated with children’s motivational and learning strategies and ultimately with academic achievement. Interaction analyses suggest that war violence was not associated with children’s academic achievement, if they had good peer relations (β = –.10, *t* = –2.09, *p* < .04) and, marginally, if parents encouraged children’s schoolwork (β =* −*.10, *t* = −1.89, *p* < .054)
Hecker et al. (2016), Tanzania	CS	409	Mean age 10.50 (6–15) years	Males and Females	Maltreatment (Corporal punishment); Children	Lifetime exposure	pediMACE	School performance and Working Memory capacity; Children	Mathematics, English, Swahili, Science, Corsi blocks	Structural equation modeling, revealed a strong relationship between harsh discipline and children’s internalizing problems (β = .47), which were in turn associated with poorer working memory (β = −.17) and school performance (β = −.17)
Jeong et al. (2020), Benin, Cambodia, Cameroon, Democratic Republic of the Congo, Honduras, Jordan, Rwanda, Senegal, Timor-Leste, Togo	CS	15,202	Mean age 47.22 (36–59) months	Males and Females	IPV; Mothers	Within the last 12 months prior to the study	CTSPC	ECD; Mothers	ECDI	Maternal stimulation mediated 1.5% of the association between IPV and ECDI *z* scores (β = –.001; *p* < .055; bias-corrected bootstrapped 95% CI: −0.002 to 0.000. While paternal stimulation mediated 3.0% of the association between IPV and ECDI z scores (β = –.002; *p* < .001; bias-corrected bootstrapped 95% CI: −0.004 to −0.001)
Julio et al. (2023), Philippines	CS	1,506	10–12 years	Males and Females	Domestic Violence: IPV- controlling behavior; Mothers	Current exposure	CLHNS	Mathematical Ability, English Reading Skills, Native Language Reading Ability, and Nonverbal Intelligence; Children	CLHNS, Philippine nonverbal intelligence local test	Mother’s vulnerability to controlling behavior decreases children’s test scores in mathematics (ATE: −3.346, *p* ≤ .01), English (ATE: −2.289, *p* ≤ .01), and nonverbal reasoning (ATE: −2.103, *p* ≤ .01). Emotional or Physical IPV was not associated with children’s test scores
Kohrt et al. (2015), Peru	CS	97	Mean 8.24 (5–11) years	Males and Females	Domestic Violence; Mothers	Lifetime exposure	WAST	Cognitive development; Children	Batería III	There was no relationship between domestic violence and cognitive development
Leyton (2020), Honduras	CS	2,256	3–4 years	Males and Females	Domestic Violence (IPV- controlling, emotional, physical and sexual violent behaviors); Mothers	Lifetime exposure	CTS	ECD; Mothers	ECDI	Children were less likely to be developmentally on track if their mothers were exposed to current partner controlling, emotional and physical violence, relative to children whose mothers had not experienced violence OR = 0.52 (0.32–0.87). Children of women who experienced current controlling, emotional, and physical violence had lower odds of being developmentally on track in the socioemotional domain of the ECDI than children of women who experienced a pattern of “no violence” 0.52 (0.32–0.86). There were no group differences in other domains, cognition (0.98 [0.32–3.00]) and literacy-numeracy (1.12 [0.53–2.36]). There were no group differences in terms of current emotional violence and being developmental on track on the ECDI (0.87 [0.61–1.25]) or in specific domains, socioemotional (0.79 [0.55–1.15]), cognition (1.91 [0.70–5.20]) and literacy-numeracy (0.66 [0.40–1.10])
Rocha et al. (2021), Brazil	CS	3,566	31.8 months	Males and Females	Domestic Violence (IPV); Mothers or head of the household	Within the last 12 months prior to the study	HITS	Child development; Mothers	ASQ-BR	The evidence for an association between emotional abuse (SMD = −0.10, 95% CI = −0.21 to −0.01), as well as physical violence (SMD = −0.08, 95% CI = −0.22 to −0.04) with personal-social domain scores was weak. There was evidence for a negative association between IPV and fine motor (SMD = −0.27, 95% CI = −0.48 to −0.06) and personal-social (SMD = −0.15, 95% CI = −0.3 to −0.01) domain scores (*p* < .05). The evidence for an association between IPV and communication scores (SMD = 0, 95% CI = −0.14 to 0.13), gross motor (SMD = −0.18, 95% CI = −0.47 to 0.09) and problem solving (SMD = −0.14, 95% CI = −0.32 to 0.05) domains (*p* > .05) was weak
Vameghi et al. (2016), Iran	CS	750	10.53 (6–18) months	Males and Females	Domestic Violence (physical, sexual, or emotional violence); Mothers	Lifetime exposure	Domestic Violence Questionnaire	Cognitive Development; Mothers	ASQ	There are no direct effects between Domestic Violence and child development. The path analysis showed that children’s development was affected indirectly by domestic violence via depression (β = −.05278)
Xing & Wang (2018), People's Republic of China	CS	150	Mean 10.83 (9–11) years	Males and Females	Maltreatment (Corporal punishment); Children	Within the last 12 months prior to the study	CTSPC	Executive Function; Mothers and Fathers	BRIEF-Parent Form	Maternal corporal punishment was positively associated with children’s behavioral regulation difficulties in the low-cortisol decline group (simple slope = 0.07, *t* = 3.19, *p* < .01) but not in the high cortisol decline group (simple slope = 0.01, *t* = 1.03, *p* > .05). The negative effect of maternal corporal punishment on children’s metacognition was greater in the low cortisol decline group (simple slope = 0.09, *t* = 4.37, *p* < .001), compared to the high cortisol decline group (simple slope = 0.04, *t* = 3.23, *p* < .01). There was also a positive association between maternal corporal punishment and children’s difficulties in global executive functions for both low and high decline cortisol groups, and the negative effect of maternal corporal punishment on global executive functions was also greater in the low cortisol decline group (simple slope = 0.09, *t* = 4.26, *p* < .001), than the high cortisol decline group (simple slope = 0.03, *t* = 2.65, *p* < .01)
Xing et al. (2019), People’s Republic of China	L	213	Mean 4.80 years	Males and Females	Maltreatment (Corporal punishment); Mothers and Fathers	Within the past 12 months, measured a year prior cognitive outcomes	CTSPC	Executive Function; Children	House and Pick the Picture, Something is the Same, Arrow, Pig, and Silly Sounds Game score	Cortisol stress reactivity level moderated the relationship between maternal (not paternal) corporal punishment and children’s executive functioning. There was a negative association between maternal corporal punishment and global executive functions in the low cortisol stress reactivity level group, β = −.42, *t* = −3.18, *p* < .05, but not in the high cortisol stress reactivity level group, β = −.13, *t* = −1.50, *p* > .05. There was also a negative association between maternal corporal punishment and children’s working memory in the low cortisol stress reactivity level group, β = −.39, *t* = −3.01, *p* < .05 but not the high cortisol stress reactivity level group, β = −.15, *t* = −1.78, *p* > .05

*Note*. ASQ = Ages and Stages Questionnaire; ASQ-BR = Ages and Stages Questionnaire–Brazil; Batería III = Woodcock-Johnson III—Spanish Version; ATE = average treatment effect; CLHNS = Cebu Longitudinal Health and Nutrition Survey; CS = cross-sectional; CTS = Conflict Tactics Scale; CTSPC = Parent-Child Conflict Tactics Scale; DP-3 = Developmental Profile III; ECD = early child development; ECDI = early child development index; HITS = Hurt, Insult, Threaten, Scream Questionnaire; IPV = intimate partner violence; L = longitudinal; SMD = standardized mean difference; WAST = Woman Abuse Screening Tool.

### Violence Exposure and Cognitive Outcomes

A majority of 71% (*n* = 12) of the reviewed studies found a relationship between violence exposure and poor cognitive outcomes in childhood. Detailed findings are described below, grouped according to type of exposure (i.e., maltreatment, IPV, and war violence).

#### Maltreatment

##### Executive Functions

**Figure 2.**
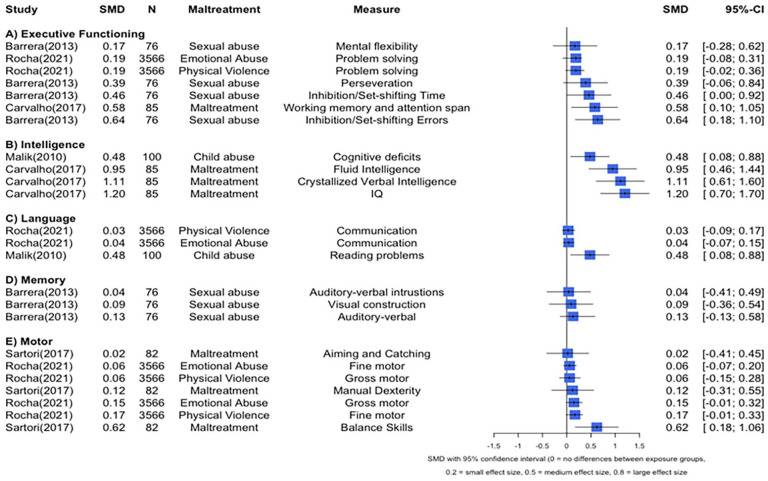
Forest plot depicting effect sizes for maltreatment and cognitive outcomes grouped by cognitive domain.

Four studies compared maltreated children to those without a history of maltreatment (as defined as sexual abuse, physical abuse, emotional abuse, physical neglect and emotional neglect) in terms of their executive functions (See Forest Plot in Figure 2A and Table 3; Barrera et al., 2013; Carvalho et al., 2017; Hecker et al., 2016; Rocha et al., 2021). A study of Columbian children aged 8 to 12 years found that children who were sexually abused had reduced inhibitory ability, indicated by more errors on the Stroop task, compared to those children who had not been sexually abused regardless of their PTSD status (Barrera et al., 2013). The Stroop task assesses cognitive flexibility and response inhibition (Lezak, 2004). It includes three parts: reading colour names in black ink, naming the ink colour of rows of stimuli to confirm understanding, and naming the ink colour of incongruent colour words (e.g., “blue” in red ink). Performance was scored based on completion time and the number of errors made. However, there were no group differences observed between children with or without a history of sexual abuse in terms of other executive functions, namely, mental flexibility, perseveration, or set-shifting time. Carvalho et al. (2017) found lower average scores on an attention span and working memory test in children with a maltreatment history compared to those without in a Brazilian sample aged 6 to 12 years. Another study with a sample of Brazilian pre-schoolers (up to 6 years of age) found that children exposed to emotional or physical abuse had lower problem-solving ability than unexposed children (Rocha et al., 2021). Conversely, Hecker et al. (2016) did not find an association between harsh discipline and working memory in a sample of Tanzanian children (mean age = 10.5 years).

**Figure 3.**
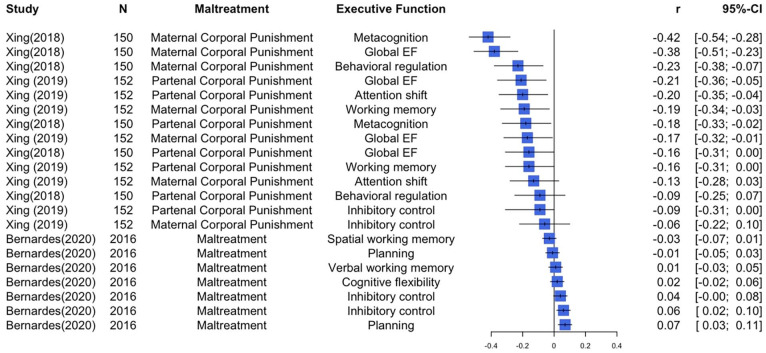
Forest plot depicting correlations between maltreatment and executive functions studies.

Three studies applied correlations to investigate the relationship between maltreatment and executive functions (See Forest Plot in Figure 3; Bernardes et al., 2020; Xing & Wang, 2018; Xing et al., 2019). Xing & Wang (2018) found weak negative correlations between paternal corporal punishment and children’s metacognition, behavioral regulation and global executive functioning in Chinese children aged 9 to 11 years. Regarding maternal corporal punishment, there were weak negative correlations with children’s behavioral regulation and metacognition, while moderate negative correlations were found with global executive functions. Another study with a sample of Chinese pre-schoolers (mean age = 4.8 years) found that paternal corporal punishment showed very weak negative correlations with children’s inhibitory control and working memory, and weak negative correlations with children’s attention shifting and global executive functions. Maternal corporal punishment was very weak and negatively correlated with all four executive function measures (Xing et al., 2019). However, in one study of Brazilian children aged 6 to 12 years, there was no strong evidence of correlations between maltreatment and executive functions, namely, spatial working memory, verbal working memory, and cognitive flexibility, including planning ability and inhibitory control—both assessed using two distinct measures each (Bernardes et al., 2020).

##### General Intelligence/Cognitive Ability

Three studies investigated the relationship between maltreatment and general cognitive functioning (See Forest Plot in Figure 2B and Table 3; Bengwasan & Bancual, 2020; Carvalho et al., 2017; Malik et al., 2010). Maltreated Brazilian children (aged 6–12 years) had lower IQ, verbal, and fluid intelligence scores than their non-maltreated counterparts (Carvalho et al., 2017). A study based on children aged 8 to 12 years from Pakistan found that abused children had higher levels of general cognitive deficits than nonabused children (Malik et al., 2010). Another study conducted in the Philippines found that abused and neglected children (aged 3–12 years) had lower intellectual ability scores than the minimum average standard score on the Developmental Profile III (DP-3; Bengwasan & Bancual, 2020).

##### Language

Three studies evaluated the relationship between maltreatment and language abilities (See Figure 2C and Table 3: Bengwasan & Bancual, 2020; Malik et al., 2010; Rocha et al., 2021). One study found that among Pakistani children (aged 8–12 years), those exposed to abuse had more reading problems than nonabused children (Malik et al., 2010). Another study conducted in the Philippines found that abused and neglected children (aged 3–12 years) had communication (expressive and receptive communication skills, including written, spoken, and gestural language) scores lower than the minimum average standard score on a development measure (Bengwasan & Bancual, 2020). In contrast, a study in Brazil did not find differences in communication ability between pre-schoolers (up to 6 years) exposed to either emotional abuse or physical abuse and those unexposed (Rocha et al., 2021).

##### Memory

Barrera et al. (2013) compared Columbian children aged 8 to 12 years who had a history of sexual abuse with those who did not (See Forest Plot in Figure 2D) and found no differences in memory abilities, including visual constructional and auditory verbal memory. Additionally, there were no group differences in terms of the number of auditory verbal intrusions during memory recall.

##### Motor Development

Two studies compared Brazilian children with a history of maltreatment to those without in terms of motor development (See Forest Plot in Figure 2E; Rocha et al., 2021; Sartori et al., 2017). In their sample of pre-schoolers, Rocha et al. (2021) found evidence to suggest a negative association between exposure to emotional abuse and gross motor but not fine motor abilities. In the same sample, they also found that exposure to physical abuse was associated with worse fine motor but not gross motor abilities. In the second study of 8- to 9-year-old children, Sartori et al. (2017) found that those who experienced maltreatment (sexual, physical, or emotional abuse, and emotional or physical neglect) had lower balance skills than children unexposed to maltreatment. However, there was no evidence to suggest that maltreated children differed from unexposed children in terms of their aiming and catching skills.

##### Social Development

A study conducted in the Philippines (Bengwasan & Bancual, 2020) found that abused and neglected children (aged 3–12 years) had socioemotional development scores lower than the minimum average standard score on a development measure. However, Rocha et al., 2021 in their sample of Brazilian pre-schoolers, did not find evidence to suggest that emotional abuse or physical violence was associated with personal-social development (see Table 3).

##### Academic Outcomes

One study did not find an association between harsh discipline and school performance in a sample of Tanzanian children (mean age = 10.5 years: Hecker et al., 2016).

#### IPV

**Figure 4.**
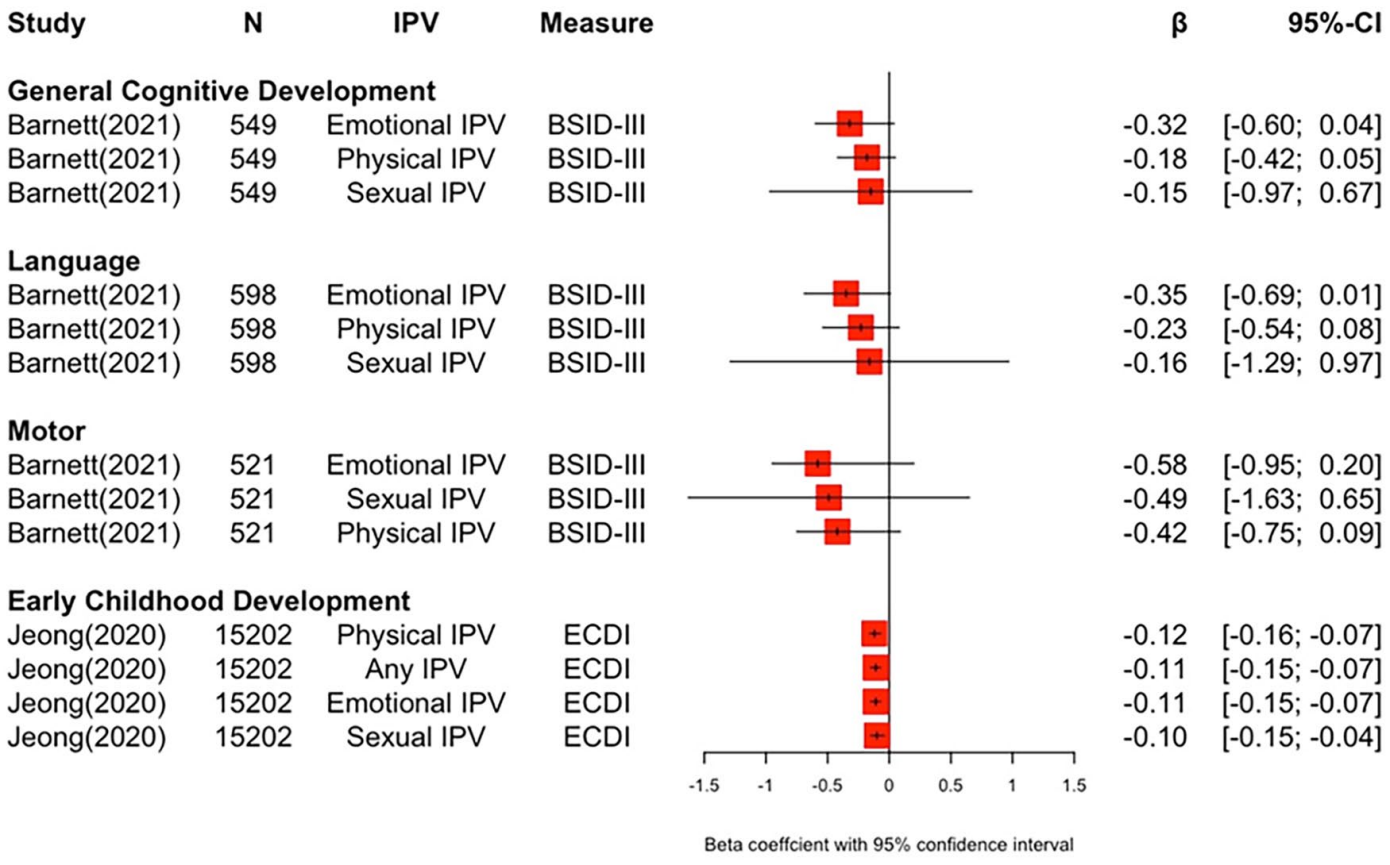
Forest plot depicting effect sizes between domestic violence and cognitive outcomes.

Five studies investigated developmental outcomes in children whose mothers experienced IPV. The majority of studies reported negative associations between domestic violence and various cognitive outcomes (Table 3; Figure 4): Maternal exposure to physical IPV compared to no exposure was associated with lower cognitive development in the fine motor and personal-social domains in Brazilian pre-schoolers (Rocha et al., 2021). In another study with pre-schoolers from 11 LMICs, a negative association was reported between maternal (physical, emotional, sexual, and any) IPV exposure and early child development scores on an index capturing cognitive, literacy, numeracy, and socioemotional development (Jeong et al., 2020). Using the same index, Leyton, (2020) found that a composite measure of maternal experience of current partner controlling behavior, emotional, and physical violence was associated with poor development compared to children whose mothers had no history of IPV in 3 to 4-year-old Honduran children. The same pattern was found in the socioemotional domain but not in other domains in this construct (cognition, literacy, and numeracy). However, there were no group differences in developmental outcomes when looking solely at maternal current partner emotional violence in the same sample. South African children aged 2 years were found to possess lower cognitive, language, and motor development scores if their mothers had been exposed to emotional IPV (Barnett et al., 2021). In the same study, maternal physical IPV was associated with lower motor scores but not cognitive or language scores. Furthermore, there was no association between maternal sexual IPV and developmental outcomes. A study conducted with children (aged 10–12 years) in the Philippines found that mothers’ experience of controlling behavior from her partner was negatively associated with children’s test scores in Mathematics, English, and nonverbal reasoning (Julio et al., 2023). In the same study, maternal physical or emotional IPV was not associated with children’s test scores.

Two other studies, however, did not find associations between maternal IPV and children’s developmental outcomes: Vameghi et al. (2016) examined the path association between maternal domestic violence experiences and the development of 6- to 18-month-old Iranian infants. They found no association between maternal IPV and communication, problem-solving, personal-social, gross, and fine motor development. Similarly, Kohrt et al. (2015) found that maternal IPV was not related to cognitive developmental functions such as verbal comprehension, fluid reasoning, and processing speed in their study with children aged 5 to 11 years in Peru.

#### War Violence

Finally, one study investigated the relationship between exposure to war and academic achievement in Palestinian children (mean age = 10.94 years) living in the Gaza Strip (Diab et al., 2018). They found no association between exposure to traumatic war experiences and children’s academic achievement including both language and math scores.

#### Mediators and Moderators

Two studies examined whether cortisol moderated the relationship between corporal punishment and executive functions in Chinese children (Xing & Wang, 2018; Xing et al., 2019) and reported similar findings (see Table 3). The first study of 9 to 11-year-olds found that the relationship between maternal corporal punishment and children’s executive functions varied based on diurnal cortisol change, but not morning or afternoon cortisol (Xing & Wang, 2018). Specifically, the relationship between maternal corporal punishment and children’s difficulties with executive functioning was stronger in a low-diurnal decline cortisol group than in a high-decline cortisol group. In the second study, cortisol stress reactivity moderated the relationship between maternal corporal punishment and children’s executive functioning in a sample of pre-schoolers (Xing et al., 2019). Specifically, past year’s exposure to maternal corporal punishment was associated with poorer global executive functions and working memory in children with low cortisol stress reactivity but not in those with high cortisol stress reactivity. In both studies, there were no moderation patterns found with paternal corporal punishment.

In a study of pre-schoolers (mean age = 47.22 months) spanning 11 LMICs, the negative relationship between IPV exposure and cognitive development was partially mediated by reduced maternal and paternal stimulation, with independent effects observed for each parent (Jeong et al., 2020). Specifically, maternal and paternal stimulation each mediated 1.5% and 3.0% of the association between IPV and early childhood development scores, respectively.

Hecker et al. (2016) investigated the relationship between harsh discipline, internalizing problems, working memory, and school performance in a sample of Tanzanian children (mean age = 10.5 years). They found an indirect association via internalizing problems between harsh discipline and poorer working memory capacity and school performance.

Internalizing problems were defined as difficulties related to emotional and social functioning, including symptoms such as peer difficulties and emotional distress. The severity of these problems was determined by evaluating the intensity and impact of depressive and anxiety-related symptoms.

Diab et al. (2018) found that the negative relationship between children’s exposure to war violence and academic achievement was mediated by parental scholastic involvement and children’s motivation and learning strategies. Specifically, high exposure to war violence was negatively associated with scholastic involvement from parents, which in turn was negatively associated with children’s motivation and learning strategies and finally with academic achievement. In the same study, interaction analyses revealed that war violence was not associated with children’s academic achievement if children had good peer relations and, marginally, if parents showed encouragement for children’s schoolwork.

Path analyses in Vameghi et al.’s (2016) study found that maternal IPV indirectly affected overall development in 6- to 18-month-old Iranian children, with maternal depression as the mediating factor. Specifically, maternal experience of domestic violence was positively associated with mothers’ depression, which in turn was negatively associated with children’s development.

### Risk of Bias

**Figure 5.**
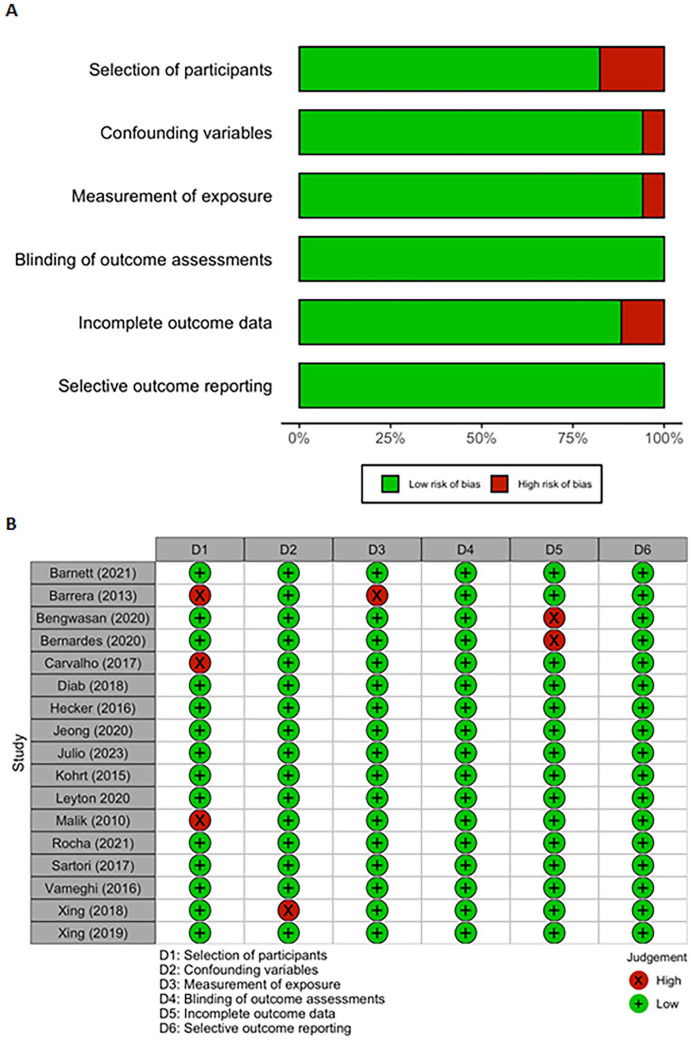
Risk of bias summary: review authors’ judgments about each risk of bias item for each included study. (A) RoBANS Risk of Bias Domain Summary; (B) RoBANS Study Risk of Bias Summary.

Figure 5A and B summarize the overall risk of bias and the risk of bias in specific domains for each included study. While the majority of studies had a low risk of bias in the domain *selection of participants* (selection biases caused by the inadequate selection of participants), approximately 20% had a high risk of bias. Approximately 15% had a high risk of bias in the domain of *incomplete outcome data* (attrition biases caused by the inadequate handling of incomplete outcome data). The domains with the lowest risk of bias were *selective outcome reporting* (reporting biases caused by the selective reporting of outcomes) and *blinding of outcome assessment* (detection biases caused by the inadequate blinding of outcome assessment). Specifically, all studies had a low risk of bias in these two domains.

**Figure 6.**
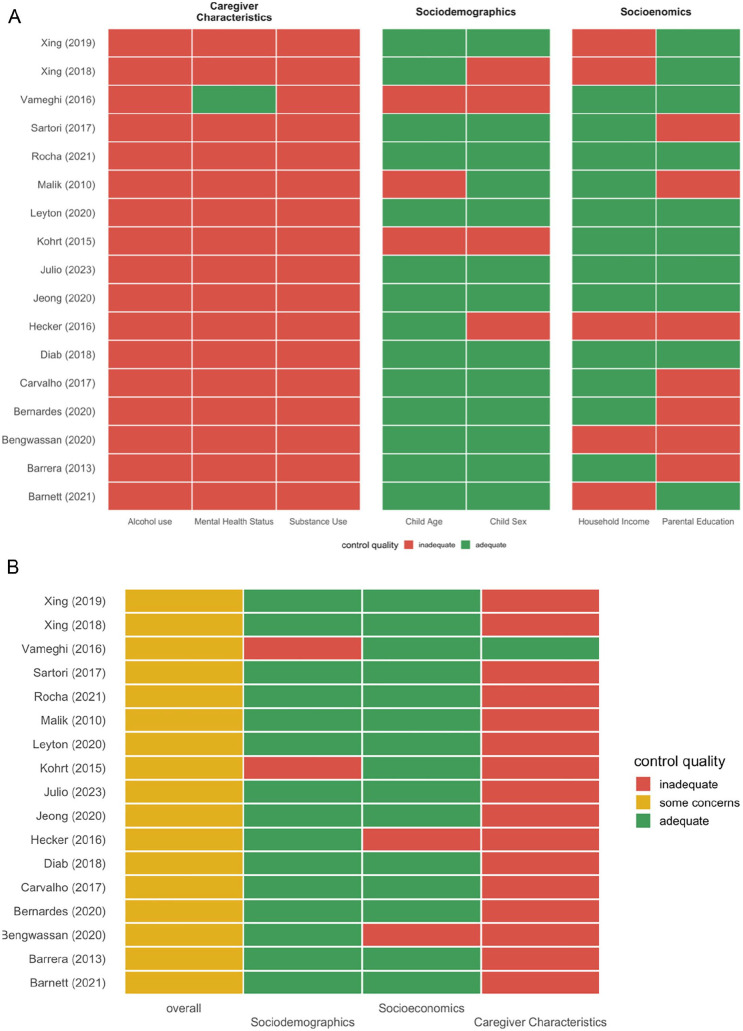
Confounder matrix illustrating confounding control of 13 studies by three confounding constructs Sociodemographics, Socioeconomics, and Caregiver Characteristics (A) and overall confounding control for each construct (B).

None of the studies had adequate control of confounding variables overall (Figure 6A and B). All the studies had “some concerns” with regards to confounding. The *Sociodemographics* and *Socioeconomics* constructs had the most adequate control, with *Child Age* and *Household Income* as the most controlled variables, respectively. The least adequate control was for the *Caregiver Characteristics* construct, with one study controlling for *Mental Health Status*.

## Discussion

We systematically reviewed and synthesized evidence on the relationship between childhood exposure to violence and cognitive outcomes in children aged 11 years and younger, residing in LMICs. A total of 17 studies which encompassed 27,643 children from 20 LMICs were examined. The majority of these studies, as expected, focused on maltreatment but IPV and war violence were also examined. The most frequently studied cognitive functions were early childhood cognitive, socio-emotional and motor development, executive functioning, general intelligence/IQ, and language ability. Overall, 71% of the reviewed studies found evidence to suggest an association between violence exposure and poor cognitive outcomes in children.

Given that this is an evolving area of research, there is currently little consensus in the literature concerning which cognitive domains are the most vulnerable to the effects of violence exposure in childhood (Su et al., 2019; Young-Southward et al., 2020). We found evidence to suggest an association between maltreatment and poor cognitive outcomes (executive functions, general intelligence, language, and aspects of cognitive development, namely, gross motor and fine motor abilities and socioemotional development) in children living in LMICs. The greatest effect size reported was for the effect of maltreatment on general intelligence (Carvalho et al., 2017), whereas the strongest correlation observed was between maternal corporal punishment on children’s metacognition (Xing & Wang, 2018). These findings largely align with those reported by Su et al. (2019) in their review investigating the influence of childhood maltreatment on later cognitive functioning in children and adults in HICs. Of note, two of their reviewed studies found that maltreated children were more likely to have reduced memory performance than non-exposed groups, whereas we found no association between childhood maltreatment and memory ability (Barrera et al., 2013). Other previous reviews reported mixed findings with regard to the association between childhood maltreatment and memory (Kavanaugh et al., 2017; Maguire et al., 2015). In the current review, these associations were only investigated by one study, whose sample size was small (*n* = 76) and possibly reduced statistical power. It is therefore possible that methodological differences between studies may have contributed to the discrepancy in findings across previous literature. As such more research is needed to investigate these associations.

The majority of reviewed studies found evidence for a negative association between maternal exposure to IPV and various aspects of children’s cognitive development (Barnett et al., 2021; Jeong et al., 2020; Leyton, 2020; Rocha et al., 2021). These findings are largely consistent with a recent review that examined the relationship between IPV and child and adolescent cognitive development primarily in HICs and found evidence to suggest that IPV in childhood was associated with poor cognitive skills in various domains (Savopoulos et al., 2023). In the current review, two other studies (Kohrt et al., 2015; Vameghi et al., 2016) did not find an association between maternal IPV and children’s cognitive development. These discrepancies may be due to methodological differences between studies, for example (Kohrt et al., 2015) had a sample size of 97, which may have limited statistical power to detect significant effects. Alternatively, discrepancies in findings may suggest the role of other intermediatory factors in these associations. Indeed, Vameghi et al. (2016) found that maternal depression mediated the relationship between maternal IPV and cognitive development in Iranian infants aged 6 to 18 months. Despite this, the current review findings, similar to those of Savopoulos et al. (2023), generally suggest the risk of poor cognitive outcomes in children whose mothers experience IPV.

The reviewed studies explored various factors that may influence the relationship between violence exposure and cognitive outcomes in childhood. Two studies found evidence to suggest that cortisol moderated the relationship between maternal corporal punishment and executive functions in Chinese children (Xing & Wang, 2018; Xing et al., 2019). These studies provide insight into the moderation effects of biological stress reactivity in influencing susceptibility to poor cognitive functioning in children exposed to maltreatment.

Indeed, it has been suggested that children’s exposure to stressful environments can lead to changes in cortisol reactivity, which has been associated with alterations to brain structure and function (Gerhardt, 2006), which, in turn, can impact cognitive functioning (Lupien et al., 2009). However, more research is needed to further understand the specific pathways in which stress and cortisol dysregulation affect cognitive development in the context of violence exposure. Other reviewed studies implicated parental factors such as parental stimulation (Jeong et al., 2020), scholastic involvement (Diab et al., 2018), and mother’s mental health (Vameghi et al., 2016), as well as child factors including academic motivation (Diab et al., 2018), internalizing problems (Hecker et al., 2016) and quality of peer relationships (Diab et al., 2018) in the associations between violence exposure and cognitive outcomes. The following reviewed studies had the most robust evidence: Jeong et al., (2020), with 15,202 children from multiple countries, found that paternal stimulation mediated 3% of the relationship between IPV and ECDI scores (β = −0.002, *p* < 0.001) after adjusting for multiple potential confounders, offering strong cross-country evidence. Rocha et al., (2021, *n* = 3,566) reported associations between IPV and fine motor (SMD = –0.27, 95% CI = –0.48 to −0.06) and personal-social development (SMD = –0.15, 95% CI = -0.30 to −0.01, *p* < 0.05). Xing et al., (2019, *n* = 213) demonstrated that maternal corporal punishment negatively impacted executive functioning in children with low cortisol stress reactivity (β = –0.42, *t* = –3.18, *p* < 0.05) using a longitudinal design that established a clear temporal relationship between corporal punishment and executive function. Indeed, researchers have highlighted the role that the early psychosocial environment such as the caregiver environment, family context, community environment as well as child characteristics play in influencing the associations between violence exposure and developmental outcomes (Berens et al., 2017). Furthermore, factors in the early psychosocial environment are particularly influential during sensitive periods of development in childhood, when specific brain regions and corresponding cognitive, and socioemotional functions are extremely responsive to environmental input (Boyce et al., 2021). This may explain the relationship between violence exposure and poor cognition in our review sample. More research is needed to explore the mechanistic role these factors play in the relationship between violence exposure and cognition as they may explain the null findings reported in some of the reviewed studies.

### Strengths and Limitations

To the authors’ knowledge, this systematic review is one of the first to examine the emerging body of research investigating violence exposure and cognition. A key strength of this review is focusing on children living in LMICs, who are known to experience disproportionately higher levels of violence than those in HICs and are widely neglected in previous literature. Regarding the diversity of the reviewed samples, the reviewed studies span 20 LMICs across Africa, Asia, and Latin America, capturing a range of geographic, cultural, and socioeconomic contexts. Participant samples were diverse in age (ranging from infancy to 12 years) and included both male and female children, offering a degree of representativeness in terms of sex and developmental stage.Furthermore, investigating these associations in children allowed us to synthesize the evidence on these relationships during childhood when exposure occurs as opposed to later. As such, the evidence suggests the risk that violence exposure poses on cognition in childhood, another strength in a research area where the majority of the literature has investigated these associations in older populations. Another strength was the inclusion of studies investigating war violence, an exposure that is particularly prevalent in the LMICs and is limited in previous literature. Furthermore, one of the strengths of our study lies in the rigorous risk of bias analyses which revealed that a number of the reviewed studies may be susceptible to attrition biases due to inadequate handling of incomplete outcome data and selection bias. We also conducted an extensive confounding control assessment which revealed that confounding control was not always adequate. The majority of the reviewed studies did not adjust for covariates related to caregiver characteristics such as mental health. Unfortunately, observational studies are notoriously vulnerable to confounding effects. By conducting our confounding control assessment, we have further highlighted this problem and encourage future research to consider confounding control as a key stage in their study design. Without adequate confounding control, the true relationship between violence exposure and cognitive outcomes may be obscured. As such it is possible that methodological differences, including differential confounding control, may contribute to discrepancies in some findings.

These findings should also be considered in the context of several limitations. The number of synthesized studies included was small, which is further indicative of the limited research being conducted in this crucial area of research. Given that some of the studies (approximately 50%) relied only on secondary reports of violence (i.e., through parents, or caregivers), exposure to violence is likely underreported in these cases, due to social desirability bias. Studies involving preschool samples usually rely on adults to report on children’s violence exposure given that young children may be unable to communicate their exposure well and researchers may want to avoid the risk of retraumatizing them. Given the differences in the methodologies of the reviewed studies, a descriptive synthesis was used which limits the generalizability of results. However, this is expected in observational research of this nature as it is frequently not feasible to conduct metanalyses where such diverse phenomena are investigated. We were able to make more meaningful contributions through a descriptive approach. We also found that some studies did not provide detailed definitions of the cognitive outcomes they assessed, thus limiting our interpretations. We further acknowledge that our search, being limited to English-language records, may have overlooked studies conducted in other languages, thus reducing the diversity of the samples reviewed. Despite these limitations, this review fills an important gap in the literature and provides the preliminary steps towards developing a systematic and comprehensive body of literature on the implications of exposure to violence and on cognitive outcomes in childhood.

Of note, of the 17 reviewed studies, only one had a longitudinal design and the rest were cross-sectional studies. This limits the determination of causality or temporality in the relationship between violence exposure and cognition in the review findings. As such, future research implementing longitudinal designs is needed in these investigations.

Given the high risk of bias in terms of incomplete outcome data and participant selection in the reviewed studies, we highlight the importance of future research in this area to pay attention to addressing missing data and the selection of participants. Methods to address selection bias include random, stratified, systematic sampling, or participant matching. Longitudinal studies should implement robust data collection methods for follow-up. Furthermore, multiple imputations and sensitive analyses should be considered to deal with missing data where appropriate.

Given that there is a lack of consensus regarding the types of measures used for violence exposure assessment, care should be taken to use well-established measures with clear definitions of exposure terms. Furthermore, validity and reliability analyses should be conducted on these measures. The following measures are recommended and were employed by some of the reviewed studies: For childhood maltreatment and abuse, widely used measures include the Adverse Childhood Experiences (ACE) Questionnaire (Anda & Felitti, 1998), the Child Trauma Questionnaire (CTQ: Bernstein et al., 1994), and the Parent-Child Conflict Tactics Scale (CTSPC: (Straus, 2017; Straus et al., 1996), all of which assess multiple forms of abuse and neglect. For IPV, tools such as the Conflict Tactics Scale (CTS : (Straus et al., 1973), Woman Abuse Screening Tool (WAST: Brown et al., 1996), and Hurt, Insult, Threaten, Scream (HITS: Sherin et al., 1998). For community violence, the ‘Things I Have Seen and Heard Scale’/ Child Exposure to Community Violence Checklist (Amaya-Jackson, 1998) and for war violence Childhood War Trauma Questionnaire (CWTQ: Macksoud, 1992).

Future studies should also consider where possible an exhaustive but appropriate list of covariates including caregiver health characteristics given that this potentially impacts findings. Best practices for covariate selection include using a theoretical framework, to identify relevant factors for the research question. Data-driven approaches, such as exploratory analyses, can highlight covariates with significant relationships to cognitive outcomes. It is also essential to document and justify the selection of covariates in statistical models to ensure transparency and reproducibility. We also suggest the use of larger samples, longitudinal, and multicenter designs in research investigating the effects of violence exposure on children’s cognitive outcomes (De Bellis et al., 2010).

**Table:** Critical findings.

• A total of 17 studies were reviewed, encompassing 27,643 children from 20 low- and middle-income countries. • Various forms of violence exposure, including maltreatment, domestic violence, and war violence, were investigated. • The most frequently studied cognitive functions were early childhood cognitive, socioemotional and motor development, executive functioning, general intelligence, and language ability. • Approximately 71% of the studies found a relationship between violence exposure and poor cognitive outcomes. • Associations were found between maltreatment and poor cognitive outcomes across all cognitive domains except memory abilities. • Exposure to maternal intimate partner violence was linked to poor early childhood cognitive, socioemotional, and motor development. • Cortisol, parental stimulation, scholastic involvement, and mental health, child academic motivation, and mental health as well as quality of peer relationships were implicated as moderators and/mediators.

**Table:** Implications for research, policy, and practice.

• More targeted research is needed in low and middle-income countries, where violence rates are high, and research is limited. • Interventions that focus on both the eradication of violence and support for children facing cognitive difficulties due to exposure to violence are needed. • Future research needs to carefully handle bias. Proper attention should be paid to address missing data and participant selection strategies. • An exhaustive and appropriate list of covariates, including caregiver health characteristics that can potentially impact findings, should be considered in future research. • There is a need for research investigating factors that mediate or moderate associations between violence exposure and cognitive outcomes in children to help our understanding of the mechanisms involved and to provide targeted interventions. • Future research to utilize larger samples, longitudinal, and multicenter designs when investigating the effects of violence exposure on children’s cognitive outcomes.

### Implications for Practice and Policy

This study highlights the need for more research investigating the effects of violence exposure on cognition in children living in LMICs, where high rates of interpersonal violence are reported (Butchart et al., 2015). Furthermore, this calls for interventions that tackle the eradication of violence as well as improve the cognitive difficulties affected children face. This is important given that cognitive problems can influence educational outcomes (Basch, 2011) and in turn alter life trajectories (Lövdén et al., 2020). There is also a need for more research investigating factors that mediate or moderate these associations between violence exposure and cognitive outcomes in children to help our understanding of the mechanisms involved and to provide targeted interventions.

Additionally, the timing of exposure plays a crucial role in its impact. Violence experienced at different life stages—such as childhood, adolescence, or adulthood—can affect individuals in distinct ways. Early exposure may influence long-term brain development (Cowell et al., 2015; Fox et al., 2010). As such research focussing on these relationships in early childhood is crucial for early intervention.

Similarly, the WHO’s INSPIRE framework recommends early interventions, including community-based Early Childhood Development programs, parental support, and routine screening in healthcare and schools. It further recommends child protection measures that enforce laws, shift harmful norms, and train teachers in non-violent discipline. The creation of safe school environments is additionally recommended. Mental health support, including trauma-informed care, is also emphasised. Moreover, the framework proposes addressing socioeconomic inequalities through poverty reduction, such as conditional cash transfers to ensure marginalised children access essential services (World Health Organization, 2016). Finally, there is a need for multi-sectoral collaboration and international support from agencies such as UNICEF and WHO to prevent violence, protect children, and promote cognitive development in LMICs.

## Supplemental Material

sj-docx-1-tva-10.1177_15248380251316232 – Supplemental material for Violence Exposure and Cognitive Outcomes Among Children in Low- and Middle-Income Countries (LMICs): A Systematic Review Supplemental material, sj-docx-1-tva-10.1177_15248380251316232 for Violence Exposure and Cognitive Outcomes Among Children in Low- and Middle-Income Countries (LMICs): A Systematic Review by Lucinda P. Tsunga, Lucy V. Hiscox, Sarah L. Halligan, Kirsten A. Donald and Abigail Fraser in Trauma, Violence, & Abuse
