# Supplementary material for: Violence Exposure and Cognitive Outcomes Among Children in Low- and Middle-Income Countries (LMICs): A Systematic Review
Source: Trauma Violence Abuse. 2025 Mar 12;27(2):407–28. doi: 10.1177/15248380251316232 (PMC12953671; doi:10.1177/15248380251316232)
Supplement: sj-docx-1-tva-10.1177_15248380251316232 – Supplemental material for Violence Exposure and Cognitive Outcomes Among Children in Low- and Middle-Income Countries (LMICs): A Systematic Review [file sj-docx-1-tva-10.1177_15248380251316232.docx]

Systematic Review of Violence Exposure and Cognitive Outcomes Among Children in Low-Middle Income Countries (LMICs) - Supplementary Material

Supplementary Table 1 DAC List of ODA Recipients Effective for reporting on 2020 flows (LMICS)

| **Least Developed Countries** | **Other Low Income Countries**  (per capita GNI <= $1 005 in 2016) | **Lower Middle Income Countries and Territories**  (per capita GNI $1 006-$3 955 in 2016) | **Upper Middle Income Countries and Territories** (per capita GNI $3 956-$12 235 in 2016) |
| --- | --- | --- | --- |
| Afghanistan | Democratic People's Republic of Korea | Armenia | Albania |
| Angola^1^ | Zimbabwe | Bolivia | Algeria |
| Bangladesh |  | Cabo Verde | Antigua and Barbuda^2^ |
| Benin |  | Cameroon | Argentina |
| Bhutan^1^ |  | Congo | Azerbaijan |
| Burkina Faso |  | Côte d'Ivoire | Belarus |
| Burundi |  | Egypt | Belize |
| Cambodia |  | El Salvador | Bosnia and Herzegovina |
| Central African Republic |  | Eswatini | Botswana |
| Chad |  | Georgia | Brazil |
| Comoros |  | Ghana | China (People's Republic of) |
| Democratic Republic of the Congo |  | Guatemala | Colombia |
| Djibouti |  | Honduras | Costa Rica |
| Eritrea |  | India | Cuba |
| Ethiopia |  | Indonesia | Dominica |
| Gambia |  | Jordan | Dominican Republic |
| Guinea |  | Kenya | Ecuador |
| Guinea-Bissau |  | Kosovo | Equatorial Guinea |
| Haiti |  | Kyrgyzstan | Fiji |
| Kiribati |  | Micronesia | Gabon |
| Lao People's Democratic Republic |  | Moldova | Grenada |
| Lesotho |  | Mongolia | Guyana |
| Liberia |  | Morocco | Iran |
| Madagascar |  | Nicaragua | Iraq |
| Malawi |  | Nigeria | Jamaica |
| Mali |  | Pakistan | Kazakhstan |
| Mauritania |  | Papua New Guinea | Lebanon |
| Mozambique |  | Philippines | Libya |
| Myanmar |  | Sri Lanka | Malaysia |
| Nepal |  | Syrian Arab Republic | Maldives |
| Niger |  | Tajikistan | Marshall Islands |
| Rwanda |  | Tokelau | Mauritius |
| Sao Tome and Principe^1^ |  | Tunisia | Mexico |
| Senegal |  | Ukraine | Montenegro |
| Sierra Leone |  | Uzbekistan | Montserrat |
| Solomon Islands^1^ |  | Viet Nam | Namibia |
| Somalia |  | West Bank and Gaza Strip | Nauru |
| South Sudan |  |  | Niue |
| Sudan |  |  | North Macedonia |
| Tanzania |  |  | Palau^2^ |
| Timor-Leste |  |  | Panama^2^ |
| Togo |  |  | Paraguay |
| Tuvalu |  |  | Peru |
| Uganda |  |  | Saint Helena |
| Vanuatu^1^ |  |  | Saint Lucia |
| Yemen |  |  | Saint Vincent and the Grenadines |
| Zambia |  |  | Samoa |
|  |  |  | Serbia |
|  |  |  | South Africa |
|  |  |  | Suriname |
|  |  |  | Thailand |
|  |  |  | Tonga |
|  |  |  | Turkey |
|  |  |  | Turkmenistan |
|  |  |  | Venezuela |
|  |  |  | Wallis and Futuna |

| **Concept 1:** Violence exposure | **Concept 2**: Children (0-11) | **Concept 2:** LMICs | **Concept 4:** Cognitive outcomes |
| --- | --- | --- | --- |
| Exposure to Violence  Adverse Childhood Experiences  Interpersonal violence  Violence  Trauma  Psychological Trauma  War-related trauma  Child Abuse  Physical Abuse  Sexual abuse  Rape  Emotional abuse  Maltreatment  Child Maltreatment  Maltreatment, Child  Maltreatment, Physical  Physical Maltreatment  Neglect  Child neglect  Spouse Abuse  Intimate Partner Violence  Parental intimate partner violence  Gender-Based Violence  Domestic violence  Community violence  Gun Violence  Bullying  Bullies  Bullied  Cyberbullying  School violence  Victimization | ***Appropriate Limiters were used for each database** | *****[**EPOC LMIC Filters 2020 were used**](https://epoc.cochrane.org/sites/epoc.cochrane.org/files/public/uploads/epoc_lmic_filters_2020_v4.docx) | **Alternate Terms:**  Cognitive/  Neurocognitive/  Cognition/  Executive Function/  Learning/  Pattern Recognition, Perception/  Psychomotor Performance/ Orientation/  Reaction Time  Memory/  Inhibition/  Language/  Problem-solving/  Neuropsychology/ Neuropsychological Tests/  Neurocognitive Disorders/  Cogniti* Disorders//  Cognitive/  Neurocognitive/  Intellectual/  Intelligence/  IQ/  Language/  visual-spatial/  memory/  motor/  psychomotor/  attention/  Executive  AND  Control/  Dysfunction/  Functioning/  Function/  deficit*/  Impairment*  Cognitive development/  Neurocognitive development |

Supplementary Table 2 Database search terms

**Database Search Strategies::**

Supplementary Table 3 Embase Search Strategy

| 1. | violence exposure.mp. or exp exposure to violence/ |
| --- | --- |
| 2. | Adverse Childhood Experiences.mp. or exp childhood adversity/ |
| 3. | exp violence/ or Interpersonal violence.mp. |
| 4. | Trauma.mp. |
| 5. | Psychological Trauma.mp. or exp psychotrauma/ |
| 6. | War-related trauma.mp. |
| 7. | Child Abuse.mp. or exp child abuse/ |
| 8. | Physical Abuse.mp. or exp physical abuse/ |
| 9. | Sexual abuse.mp. or exp sexual abuse/ |
| 10. | exp attempted rape/ or exp acquaintance rape/ or exp rape/ or Rape.mp. |
| 11. | Emotional abuse.mp. or exp emotional abuse/ |
| 12. | Maltreatment.mp. |
| 13. | Child Maltreatment.mp. |
| 14. | Physical Maltreatment.mp. |
| 15. | Neglect.mp. or exp emotional neglect/ or exp child neglect/ or exp neglect/ |
| 16. | Spouse Abuse.mp. or exp partner violence/ |
| 17. | Intimate Partner Violence.mp. |
| 18. | Parental intimate partner.mp. |
| 19. | exp gender based violence/ or Gender-Based Violence.mp. or exp domestic violence/ |
| 20. | exp dating violence/ |
| 21. | Community violence.mp. |
| 22. | Gun Violence.mp. or exp gun violence/ |
| 23. | exp sexual bullying/ or exp bullying/ or Bullying.mp. |
| 24. | bullies.mp. |
| 25. | Bullied.mp. |
| 26. | Cyberbullying.mp. or cyberbullying/ |
| 27. | School violence.mp. |
| 28. | Victimisation.mp. |
| 29. | Victimization.mp. |
| 30. | 1 or 2 or 3 or 4 or 5 or 6 or 7 or 8 or 9 or 10 or 11 or 12 or 13 or 14 or 15 or 16 or 17 or 18 or 19 or 20 or 21 or 22 or 23 or 24 or 25 or 26 or 27 or 28 or 29 |
| 31. | (afghanistan or albania or algeria or american samoa or angola or "antigua and barbuda" or antigua or barbuda or argentina or armenia or armenian or aruba or azerbaijan or bahrain or bangladesh or barbados or republic of belarus or belarus or byelarus or belorussia or byelorussian or belize or british honduras or benin or dahomey or bhutan or bolivia or "bosnia and herzegovina" or bosnia or herzegovina or botswana or bechuanaland or brazil or brasil or bulgaria or burkina faso or burkina fasso or upper volta or burundi or urundi or cabo verde or cape verde or cambodia or kampuchea or khmer republic or cameroon or cameron or cameroun or central african republic or ubangi shari or chad or chile or china or colombia or comoros or comoro islands or iles comores or mayotte or democratic republic of the congo or democratic republic congo or congo or zaire or costa rica or "cote d’ivoire" or "cote d’ ivoire" or cote divoire or cote d ivoire or ivory coast or croatia or cuba or cyprus or czech republic or czechoslovakia or djibouti or french somaliland or dominica or dominican republic or ecuador or egypt or united arab republic or el salvador or equatorial guinea or spanish guinea or eritrea or estonia or eswatini or swaziland or ethiopia or fiji or gabon or gabonese republic or gambia or "georgia (republic)" or georgian or ghana or gold coast or gibraltar or greece or grenada or guam or guatemala or guinea or guinea bissau or guyana or british guiana or haiti or hispaniola or honduras or hungary or india or indonesia or timor or iran or iraq or isle of man or jamaica or jordan or kazakhstan or kazakh or kenya or "democratic people’s republic of korea" or republic of korea or north korea or south korea or korea or kosovo or kyrgyzstan or kirghizia or kirgizstan or kyrgyz republic or kirghiz or laos or lao pdr or "lao people's democratic republic" or latvia or lebanon or lebanese republic or lesotho or basutoland or liberia or libya or libyan arab jamahiriya or lithuania or macau or macao or republic of north macedonia or macedonia or madagascar or malagasy republic or malawi or nyasaland or malaysia or malay federation or malaya federation or maldives or indian ocean islands or indian ocean or mali or malta or micronesia or federated states of micronesia or kiribati or marshall islands or nauru or northern mariana islands or palau or tuvalu or mauritania or mauritius or mexico or moldova or moldovian or mongolia or montenegro or "montenegro (republic)" or morocco or ifni or mozambique or portuguese east africa or myanmar or burma or namibia or nepal or netherlands antilles or nicaragua or niger or nigeria or oman or muscat or pakistan or panama or papua new guinea or new guinea or paraguay or peru or philippines or philipines or phillipines or phillippines or poland or "polish people's republic" or portugal or portuguese republic or puerto rico or romania or russia or russian federation or ussr or soviet union or union of soviet socialist republics or rwanda or ruanda or samoa or pacific islands or polynesia or samoan islands or navigator island or navigator islands or "sao tome and principe" or saudi arabia or senegal or serbia or seychelles or sierra leone or slovakia or slovak republic or slovenia or melanesia or solomon island or solomon islands or norfolk island or norfolk islands or somalia or south africa or south sudan or sri lanka or ceylon or "saint kitts and nevis" or "st. kitts and nevis" or saint lucia or "st. lucia" or "saint vincent and the grenadines" or saint vincent or "st. vincent" or grenadines or sudan or suriname or surinam or dutch guiana or netherlands guiana or syria or syrian arab republic or tajikistan or tadjikistan or tadzhikistan or tadzhik or tanzania or tanganyika or thailand or siam or timor leste or east timor or togo or togolese republic or tonga or "trinidad and tobago" or trinidad or tobago or tunisia or "turkey (republic)" or turkey or turkmenistan or turkmen or uganda or ukraine or uruguay or uzbekistan or uzbek or vanuatu or new hebrides or venezuela or vietnam or viet nam or middle east or west bank or gaza or palestine or yemen or yugoslavia or zambia or zimbabwe or northern rhodesia or global south or africa south of the sahara or "sub saharan africa" or subsaharan africa or africa, central or central africa or africa, northern or north africa or northern africa or magreb or maghrib or sahara or africa, southern or southern africa or africa, eastern or east africa or eastern africa or africa, western or west africa or western africa or west indies or indian ocean islands or caribbean region or caribbean islands or caribbean or central america or latin america or "south and central america" or south america or asia, central or central asia or asia, northern or north asia or northern asia or asia, southeastern or southeastern asia or south eastern asia or southeast asia or south east asia or asia, western or western asia or europe, eastern or east europe or eastern europe or developing country or developing countries or developing nation? or developing population? or developing world or less developed countr* or less developed nation? or less developed population? or less developed world or lesser developed countr* or lesser developed nation? or lesser developed population? or lesser developed world or under developed countr* or under developed nation? or under developed population? or under developed world or underdeveloped countr* or underdeveloped nation? or underdeveloped population? or underdeveloped world or middle income countr* or middle income nation? or middle income population? or low income countr* or low income nation? or low income population? or lower income countr* or lower income nation? or lower income population? or underserved countr* or underserved nation? or underserved population? or underserved world or under served countr* or under served nation? or under served population? or under served world or deprived countr* or deprived nation? or deprived population? or deprived world or poor countr* or poor nation? or poor population? or poor world or poorer countr* or poorer nation? or poorer population? or poorer world or developing econom* or less developed econom* or lesser developed econom* or under developed econom* or underdeveloped econom* or middle income econom* or low income econom* or lower income econom* or low gdp or low gnp or low gross domestic or low gross national or lower gdp or lower gnp or lower gross domestic or lower gross national or lmic or lmics or third world or lami countr* or transitional countr* or emerging economies or emerging nation?).mp. [mp=title, abstract, heading word, drug trade name, original title, device manufacturer, drug manufacturer, device trade name, keyword, floating subheading word, candidate term word] |
| 32. | 30 and 31 |
| 33. | cognitive outcomes.mp. |
| 34. | Cognition.mp. or exp cognition assessment/ or exp cognition/ |
| 35. | Executive Function.mp. or exp executive function/ |
| 36. | Higher Nervous Activity.mp. or exp central nervous system function/ |
| 37. | Learning.mp. or exp learning test/ or exp learning/ or exp learning disorder/ or exp "learning and memory test"/ |
| 38. | Pattern Recognition.mp. or exp pattern recognition/ |
| 39. | Perception Visual.mp. |
| 40. | exp perception/ or Perception.mp. or exp visual perception test/ |
| 41. | exp thinking impairment/ or exp concrete thinking/ or exp conceptual thinking/ or Thinking.mp. or exp critical thinking/ or exp abstract thinking/ or exp abnormal thinking/ or exp thinking/ |
| 42. | Volition.mp. |
| 43. | Psychomotor Performance.mp. or exp psychomotor performance/ |
| 44. | exp visual orientation/ or exp spatial orientation/ or Orientation.mp. or exp orientation/ |
| 45. | exp retrospective memory/ or exp memory disorder/ or exp episodic memory/ or exp non-spatial memory test/ or exp memory bias/ or exp auditory memory/ or exp prospective memory/ or exp memory assessment/ or exp verbal memory test/ or exp nonverbal memory test/ or exp memory test/ or exp memory consolidation/ or exp long term memory/ or exp working memory network/ or exp reference memory/ or exp sensory memory/ or exp implicit memory/ or exp working memory/ or exp "test of memory malingering"/ or exp Wechsler memory scale/ or exp associative memory/ or exp procedural memory/ or exp declarative memory/ or Memory.mp. or exp spatial memory test/ or exp false memory/ or exp "learning and memory test"/ or exp word memory test/ or exp memory/ or exp spatial memory/ or exp explicit memory/ or exp semantic memory/ or exp short term memory/ or exp visual memory/ or exp verbal memory/ or exp context-dependent memory/ |
| 46. | exp "inhibition (psychology)"/ or Inhibition.mp. |
| 47. | exp developmental language disorder/ or exp language ability/ or exp language disability/ or exp "speech and language rehabilitation"/ or exp language processing/ or exp language delay/ or Language.mp. or exp "speech and language assessment"/ or exp language/ or exp language development/ or exp language test/ or exp preschool language scale/ or exp written language/ |
| 48. | Problem solving.mp. or exp problem solving/ |
| 49. | exp neuropsychological test/ or Neuropsychology.mp. or exp neuropsychology/ or exp cognitive defect/ |
| 50. | Neuropsychological Tests.mp. or exp neuropsychological test/ |
| 51. | exp intelligence/ or General Intelligence.mp. |
| 52. | Intelligent Quotient.mp. or exp intelligence quotient/ |
| 53. | IQ.mp. |
| 54. | Neurocognitive Disorders.mp. |
| 55. | exp transient global amnesia/ or exp anterograde amnesia/ or exp retrograde amnesia/ or exp amnesia/ or Amnesia.mp. or exp dissociative amnesia/ |
| 56. | Cognitive Disorders.mp. or exp cognitive defect/ |
| 57. | 33 or 34 or 35 or 36 or 37 or 38 or 39 or 40 or 41 or 42 or 43 or 44 or 45 or 46 or 47 or 48 or 49 or 50 or 51 or 52 or 53 or 54 or 55 or 56 |
| 58. | 32 and 57 |
| 59. | limit 58 to (abstracts and human and english language and (infant or child or preschool child <1 to 6 years> or school child <7 to 12 years>)) |
| 60. | limit 59 to full text |

Supplementary Table 4 studies that might appear to meet the inclusion criteria, but which were excluded

|  | Study | Title | Exclusion Reason |
| --- | --- | --- | --- |
| 1 | (Ayoub et al., 2006) | Cognitive and emotional differences in young maltreated children: a translational application of dynamic skill theory. | Study conducted in the USA- Not LMIC |
| 2 | (Buckner & Kim, 2012) | Mobile innovations, executive functions, and educational developments in conflict zones: A case study from Palestine. | Violence exposure is indirect |
| 3 | (Liu et al., 2017) | Relationships of bullying involvement with intelligence, attention, and executive function in children and adolescents with attention-deficit/hyperactivity disorder | Children already diagnosed with ADHD |
| 4 | (MacDonald, 2008) | Attention, memory, and executive functioning in children with posttraumatic stress symptomatology. | Study conducted in the USA- Not LMIC |
| 5 | (Mougrabi-Large, 2016) | War, trauma, and cognition of Palestinian children. | Violence exposure is indirect |
| 6 | (Naudé et al., 2007) | Executive emotional system disruption as causal agent in frontal lobishness among abused children. | Ages 8 - 16, analyses didn't separate age groups |
| 7 | (Punamäki et al., 2011) | Who are the resilient children in conditions of military violence? Family- and child-related factors in a Palestinian community sample. | Ages 6 - 16, analyses didn't separate age groups |
| 8 | (Rosales et al., 2019) | Behavior change communication model enhancing parental practices for improved early childhood growth and development outcomes in rural Armenia - A quasi-experimental study. | No violence exposure |

References

Ayoub, C. C., O’Connor, E., Rappolt-Schlichtmann, G., Fischer, K. W., Rogosch, F. A., Toth, S. L., & Cicchetti, D. D. (2006). Cognitive and emotional differences in young maltreated children: A translational application of dynamic skill theory. *Development and Psychopathology*, *18*(3), 679–706. https://doi.org/10.1017/S0954579406060342

Buckner, E., & Kim, P. (2012). Mobile innovations, executive functions, and educational developments in conflict zones: A case study from Palestine. *Educational Technology Research and Development*, *60*(1), 175–192. https://doi.org/10.1007/S11423-011-9221-6/TABLES/6

Liu, T.-L., Guo, N.-W., Hsiao, R. C., Hu, H.-F., & Yen, C.-F. (2017). Relationships of bullying involvement with intelligence, attention, and executive function in children and adolescents with attention-deficit/hyperactivity disorder. *Research in Developmental Disabilities*, *70*, 59–66. https://doi.org/10.1016/j.ridd.2017.08.004

MacDonald, H. Z. (2008). Attention, memory, and executive functioning in children with posttraumatic stress symptomatology [dissertation]. In *Boston University, 2008. 117 pp.* https://bris.idm.oclc.org/login?url=https://www.proquest.com/dissertations-theses/attention-memory-executive-functioning-children/docview/42465200/se-2?accountid=9730

Mougrabi-Large, R. (2016). War, trauma, and cognition of Palestinian children. In *ProQuest Dissertations and Theses*. https://bris.idm.oclc.org/login?url=https://www.proquest.com/dissertations-theses/war-trauma-cognition-palestinian-children/docview/1756243348/se-2?accountid=9730

Naudé, H., du Preez, C. S., & Pretorius, E. (2007). Executive emotional system disruption as causal agent in frontal lobishness among abused children. *Http://Dx.Doi.Org/10.1080/0300443032000153615*, *174*(5), 437–460. https://doi.org/10.1080/0300443032000153615

Punamäki, R. L., Qouta, S., Miller, T., & El-Sarraj, E. (2011). Who Are the Resilient Children in Conditions of Military Violence? Family- and Child-Related Factors in a Palestinian Community Sample. *APA*, *17*(4), 389–416. https://doi.org/10.1080/10781919.2011.610722

Rosales, A., Sargsyan, V., Abelyan, K., Hovhannesyan, A., Ter-Abrahanyan, K., Jillson, K. Q., & Cherian, D. (2019). Behavior change communication model enhancing parental practices for improved early childhood growth and development outcomes in rural Armenia – A quasi-experimental study. *Preventive Medicine Reports*, *14*, 100820. https://doi.org/10.1016/J.PMEDR.2019.100820
